# Supplementary material for: Integration of human pancreatic islet genomic data refines regulatory mechanisms at Type 2 Diabetes susceptibility loci
Source: eLife. 2018 Feb 7;7:e31977. doi: 10.7554/eLife.31977 (PMC5828664; doi:10.7554/eLife.31977)
Supplement: Figure 3—source data 1. — For each annotation the data source and the log2 Fold Enrichment (log2FE) in T2D is shown. 95% Confidence Intervals (CI) for log2FE are shown in brackets and significantly enriched states are highlighted in bold (lower CI limit >0). [file elife-31977-fig3-data1.docx]

| **Regulatory**  **States** | **Source** | **single T2D**  **enrichment (CI)** |
| --- | --- | --- |
| **ATAC** | Open chromatin | **3.4 (2.6 to 4)** |
| **LMR** | WGBS | **3.2 (2.3 to 3.9)** |
| **UMR** | WGBS | 1.4 (-0.6 to 2.5) |
| **PMD** | WGBS | -0.8 (-1.7 to -0.1) |
| **dDMR** | WGBS | -24.6 (-44.6 to 3.7) |
| **All Strong Enhancer** | ChIP-only | **2.9 (2.1 to 3.5)** |
| **All Genic Enhancer** | ChIP-only | **2.7 (1.6 to 3.5)** |
| **All TSS upstream** | ChIP-only | **2.4 (0.9 to 3.4)** |
| **All Active Promoter** | ChIP-only | **2.2 (0.8 to 3.2)** |
| **All Weak Enhancer** | ChIP-only | **1.3 (0.2 to 2.1)** |
| **CDS** | Protein-coding | **2.6 (1.2 to 3.5)** |
| **CONS** | Conserved Sequence | **2.1 (1.1 to 2.9)** |
