# Supplementary material for: Integration of human pancreatic islet genomic data refines regulatory mechanisms at Type 2 Diabetes susceptibility loci
Source: eLife. 2018 Feb 7;7:e31977. doi: 10.7554/eLife.31977 (PMC5828664; doi:10.7554/eLife.31977)
Supplement: Figure 3—source data 2. — For each annotation the single feature and joint-model log2 Fold Enrichment (log2FE) in T2D is shown. 95% Confidence Intervals (CI) for log2FE are shown in brackets. In addition, the LRT statistic and P-value of a nested joint-model excluding a given annotation is shown. [file elife-31977-fig3-data2.docx]

|  | **Single state Enrichment (CI)** | **Joint model**  **Enrichment (CI)** | **LRT statistic**  **(Chi-square)** | **LRT**  **P-value** |
| --- | --- | --- | --- | --- |
| **ATAC-seq**  **open chromatin** | 3.4 (2.6 to 4) | 2.6 (2.0 to 3.3) | 31.0 | 2.6E-08 |
| **LMR** | 3.2 (2.3 to 3.9) | 1.1 (0.1 to 1.8) | 4.2 | 0.04 |
| **ChIP-only**  **Gene Enhancer** | 2.7 (1.6 to 3.5) | 2.8 (1.7 to 3.6) | 14.3 | 1.6E-04 |
| **ChIP-only**  **Strong Enhancer** | 2.9 (2.1 to 3.5) | 1.8 (0.9 to 2.4) | 11.1 | 8.8E-04 |
| **ChIP-only**  **Weak Enhancer** | 1.3 (0.2 to 2.1) | 1.7 (0.8 to 2.4) | 8.9 | 0.003 |
| **CDS** | 2.6 (1.2 to 3.5) | 2.1 (1.0 to 3.0) | 10.3 | 0.001 |
