# Supplementary material for: Integration of human pancreatic islet genomic data refines regulatory mechanisms at Type 2 Diabetes susceptibility loci
Source: eLife. 2018 Feb 7;7:e31977. doi: 10.7554/eLife.31977 (PMC5828664; doi:10.7554/eLife.31977)
Supplement: Figure 3—source data 3. — For each annotation the single feature and joint-model log2 Fold enrichment (log2FE) in FG is shown. 95% Confidence Intervals (CI) for log2FE are shown in brackets. In addition, the LRT statistic and P-value of a nested joint-model excluding a given annotation is shown. [file elife-31977-fig3-data3.docx]

|  | **Single state Enrichment (CI)** | **Joint model**  **Enrichment (CI)** | **LRT statistic**  **(Chi-square)** | **LRT**  **P-value** |
| --- | --- | --- | --- | --- |
| **ChIP-only**  **Strong Enhancer** | 3.7 (2.5 to 4.8) | 3.4 (2.2 to 4.5) | 11.4 | 7.4E-04 |
| **ChIP-only**  **TSS upstream** | 3.3 (1.6 to 4.6) | 3.1 (1.5 to 4.3) | 7.3 | 0.007 |
| **ATAC-seq**  **open chromatin** | 4.0 (2.8 to 5.1) | 1.1 (-0.4 to 2.3) | 1.7 | 0.20 |
| **LMR** | 3.9 (2.3 to 5.1) | 1 (-0.8 to 2.3) | 1.4 | 0.23 |
