# Supplementary material for: Integration of human pancreatic islet genomic data refines regulatory mechanisms at Type 2 Diabetes susceptibility loci
Source: eLife. 2018 Feb 7;7:e31977. doi: 10.7554/eLife.31977 (PMC5828664; doi:10.7554/eLife.31977)
Supplement: Figure 4—source data 1. — For each set of annotations used the median segment top variant PPA (thigher values indicate better performance), the median segment 99% credible set size (lower values indicate better performance) and the number of significant segments (higher number indicates better performance) is shown. Significant loci were defined solely on a combined segmental PPA of at least 0.90. [file elife-31977-fig4-data1.docx]

| **Annotation** | **Median Max variant PPA** | **Median 99%**  **credible set size** | **No of significant**  **segments (PPA>0.9)** |
| --- | --- | --- | --- |
| **ChIP-only** | 0.310 | 23 | 51 |
| **ChIP+Meth** | 0.267 | 23 | 50 |
| **ChIP+ATAC** | 0.391 | 18 | 48 |
| **ChIP+ATAC+Meth** | 0.386 | 17 | 50 |
| **ATAC-only** | 0.384 | 19 | 49 |
| **LMR-only** | 0.282 | 30 | 48 |
