# Supplementary material for: Integration of human pancreatic islet genomic data refines regulatory mechanisms at Type 2 Diabetes susceptibility loci
Source: eLife. 2018 Feb 7;7:e31977. doi: 10.7554/eLife.31977 (PMC5828664; doi:10.7554/eLife.31977)
Supplement: Transparent reporting form [file elife-31977-transrepform.docx]

***eLife’s* transparent reporting form**

We encourage authors to provide detailed information *within their submission* to facilitate the interpretation and replication of experiments. Authors can upload supporting documentation to indicate the use of appropriate reporting guidelines for health-related research (see [EQUATOR Network](http://www.equator-network.org/%20)), life science research (see the [BioSharing Information Resource](https://biosharing.org/)), or the [ARRIVE guidelines](http://www.plosbiology.org/article/info:doi/10.1371/journal.pbio.1000412) for reporting work involving animal research. Where applicable, authors should refer to any relevant reporting standards documents in this form.

If you have any questions, please consult our Journal Policies and/or contact us: [editorial@elifesciences.org](mailto:editorial@elifesciences.org).

**Sample-size estimation**

- You should state whether an appropriate sample size was computed when the study was being designed
- You should state the statistical method of sample size computation and any required assumptions
- If no explicit power analysis was used, you should describe how you decided what sample (replicate) size (number) to use

Please outline where this information can be found within the submission (e.g., sections or figure legends), or explain why this information doesn’t apply to your submission:

As described in detail in the introduction and discussion section (as well as the cover letter) access to human pancreatic islet tissue has been limited to date. Access is predominantly via islets isolated from pancreata recovered from cadaveric donors. This tissue type is underrepresented in community epigenome projects such as Encode and Epigenome Roadmap.

The number of human islet samples included in this study to characterise the islet methylome (WGBS non-diabetic samples n=10) and open chromatin status (ATAC-seq non-diabetic samples n=17) exceeds that reported in other previously published human islet datasets. This includes the recent studies focussing on islet chromatin (Varshney et al 2017 ATAC-seq n=2, Roman et al 2017 H3K27ac ChIP-seq n=1) and DNA methylation status (Volker et al 2017 WGBS non-diabetic samples n=8). Specifically, Roman et al 2017 used a single ChIP-seq sample to investigate H3K27ac allelic imbalance at the *ADCY5* locus while Varshney et al used 2 ATAC-seq samples to characterise open chromatin allelic imbalance signals in transcription factor footprints (TFBS). Our study extends these studies by investigating open chromatin status and allelic imbalance in human islet samples derived from 17 non-diabetic donors. To reduce multiple testing burden associated with a genome-wide chromatin quantitative trait loci analysis, we focused on heterozygous variants associated with Type 2 Diabetes genetic risk and located in human islet regulatory elements.

In this kind of genome-wide genomic study, one is in effect testing many millions of different hypotheses in parallel, so “traditional” sample size calculations based on a single hypothesis are not appropriate. As we demonstrate the sample sizes deployed here allow us to detect some positive signals to robust levels of significance, but there is no doubt that even larger sample sizes would recover additional samples.

**Replicates**

- You should report how often each experiment was performed
- You should include a definition of biological versus technical replication
- The data obtained should be provided and sufficient information should be provided to indicate the number of independent biological and/or technical replicates
- If you encountered any outliers, you should describe how these were handled
- Criteria for exclusion/inclusion of data should be clearly stated
- High-throughput sequence data should be uploaded before submission, with a private link for reviewers provided (these are available from both GEO and ArrayExpress)

Please outline where this information can be found within the submission (e.g., sections or figure legends), or explain why this information doesn’t apply to your submission:

Throughout the abstract, results and methods sections as well as figure legends, we have provided detailed information on the number of replicates and sample donors. Ethical and consent rules restrict release of the individual level data to controlled access repositories. We have found it challenging in the past to provide private access to reviewers since reviewers would likely require to obtain Institutional review board (IRB) approval to access the data (or go through EGA approval). In line with the ethics requirements, we are preparing the relevant human sequencing raw and summary level data for release via controlled access routes (EGA study accession number: EGAS00001002592). At publication the data will be available for all bona fide investigators via EGA. Summary level/source data files that do not provide information specific to individual donors will be made available as described below.

**Statistical reporting**

- Statistical analysis methods should be described and justified
- Raw data should be presented in figures whenever informative to do so (typically when N per group is less than 10)
- For each experiment, you should identify the statistical tests used, exact values of N, definitions of center, methods of multiple test correction, and dispersion and precision measures (e.g., mean, median, SD, SEM, confidence intervals; and, for the major substantive results, a measure of effect size (e.g., Pearson's r, Cohen's d)
- Report exact p-values wherever possible alongside the summary statistics and 95% confidence intervals. These should be reported for all key questions and not only when the p-value is less than 0.05.

Please outline where this information can be found within the submission (e.g., sections or figure legends), or explain why this information doesn’t apply to your submission:

Information about statistical tests, multiple testing corrections, P-values and confidence intervals is provided as part of the main text in the relevant results and methods sections. Additional information is provided in figure legends and tables.

(For large datasets, or papers with a very large number of statistical tests, you may upload a single table file with tests, Ns, etc., with reference to sections in the manuscript.)

**Group allocation**

- Indicate how samples were allocated into experimental groups (in the case of clinical studies, please specify allocation to treatment method); if randomization was used, please also state if restricted randomization was applied
- Indicate if masking was used during group allocation, data collection and/or data analysis

Please outline where this information can be found within the submission (e.g., sections or figure legends), or explain why this information doesn’t apply to your submission:

No specific grouping allocations were used in this study.

**Additional data files (“source data”)**

- We encourage you to upload relevant additional data files, such as numerical data that are represented as a graph in a figure, or as a summary table
- Where provided, these should be in the most useful format, and they can be uploaded as “Source data” files linked to a main figure or table
- Include model definition files including the full list of parameters used
- Include code used for data analysis (e.g., R, MatLab)
- Avoid stating that data files are “available upon request”

Please indicate the figures or tables for which source data files have been provided:

As described above, human genomic datasets will be made available through controlled access routes to comply with ethics and consent rules. In addition, we have included in the submission three source data bed files providing the following human pancreatic islet summary level information that is not specific to any individual donors: * bed file of human pancreatic islet chromatin states (human_islet_chromatin_states.bed)

* bed file of human pancreatic WGBS UMR and LMR regulatory regions (human_islet_LMRs_UMRs.bed) and
* bed file of ATAC-seq open chromatin peaks (human_islet_chromatin_peaks.bed).
